# Is parent education a factor in identifying autism/takiwātanga in an ethnic cohort of Pacific children in Aotearoa, New Zealand? A national cross-sectional study using linked administrative data

**DOI:** 10.1177/13623613231217800

**Published:** 2023-12-28

**Authors:** Jesse Kokaua, Betty Kolose-Pulefolau, Troy Ruhe, Faith Aldridge, Siale Foliaki, Liam Kokaua, Talai Mapusua, Joanne Dacombe, Rosalina Richards, Russell Blakelock, Nicholas Bowden

**Affiliations:** 1 University of Otago, New Zealand; 2 Pasifika Autism Support Group, New Zealand; 3 Te Whatu Ora Counties Manukau, New Zealand; 4 Independent Researcher, New Zealand; 5 Te Whatu Ora Canterbury Rohe, New Zealand

**Keywords:** autism, Pacific ethnicity, parental education, population data

## Abstract

Studies have found early detection of autism is protective for an individual and their family. Fewer Pacific children are diagnosed with autism compared with non-Pacific children, and those who have significantly higher needs, suggesting an undercount of those diagnosed. The purpose of this study was to explore any association between parental education and autism in Pacific children living in Aotearoa, New Zealand. This was a national cross-sectional study using data from New Zealand’s Integrated Data Infrastructure, including Pacific and non-Māori non-Pacific children in the 2013 Census. A novel method was employed to capture autism, while years of parents’ education was estimated from their highest attained qualification level. Apposite regression models were used to analyse the relationship between parental education and autism. We found that autism was identified in 1.1% of Pacific children compared with 1.6% of non-Māori, non-Pacific children. A year of education among parents of Pacific children was associated with increased identification of autism (odds ratio = 1.10; 95% confidence interval = 1.06–1.15). The findings show education plays a positive role in receiving a diagnosis for Pacific autistic children but suggest a systemic failure to support Pacific parents to navigate existing health systems for themselves or their children.

**Lay abstract:**

Previous studies of autism in Aotearoa, New Zealand, suggest that fewer Pacific children receive an autism diagnosis compared to European children. This study aimed to explore if formal education qualification of parents is related to receiving an autism diagnosis for their Pacific child. Our findings show that autism was identified in 1.1% of Pacific children compared with 1.6% among non-Māori, non-Pacific children. Parents with higher levels of education were more likely to receive an autism diagnosis for their Pacific child. While the study findings indicate education plays a positive role in receiving a diagnosis for autistic children, they suggest a systemic failure of supporting Pacific parents and communities to navigate the health and education systems that exist in Aotearoa, New Zealand.

## Background

Research indicates that early detection of autism is in the best interest of both the individual and their family since earlier intervention supports positive outcomes on later skills (Magiati et al., 2014; Whitehouse et al., 2021). However, receiving an autism diagnosis can be a difficult and prolonged process that requires a high level of determination from parents or caregivers. In Aotearoa, New Zealand (Aotearoa/NZ), it takes on average approximately three and a half years from the first instance of documented parent concern of possible autism for their child, at a mean age of 3.2 years, to receiving an autism diagnosis, 6.6 years (Eggleston et al., 2019). Moreover, parents report high levels of dissatisfaction with the diagnostic process in Aotearoa/NZ. Given the time and persistence required to obtain an autism diagnosis, it has been hypothesised that family resourcing including socioeconomic status and parents’ education levels may be associated with inequities in the diagnostic process (Dickerson et al., 2017; Durkin et al., 2010; Kelly et al., 2017; Thomas et al., 2012).

Current research provides conflicting evidence on the association between autism diagnosis and socioeconomic factors. Studies from the United States have historically found that autism rates are higher among children of higher socioeconomic status (Dickerson et al., 2017; Durkin et al., 2010; Fountain et al., 2011; Thomas et al., 2012) including associations between higher levels of parental education and receiving an autism diagnosis. However, more recently, the Autism and Developmental Disabilities Monitoring (ADDM) Network has shown autism prevalence is relatively stable in relation to income level (Maenner et al., 2023). A recent large-scale study from the United Kingdom found that autism was associated with socioeconomic disadvantage (Roman-Urrestarazu et al., 2021). However, another UK-based study found that education attainment level among mothers was positively associated with receiving an autism diagnosis for their child (Kelly et al., 2019). Research from other parts of the world have also established conflicting findings (Larsson et al., 2005; Rai et al., 2012). Therefore, it appears that the link between autism and socioeconomic status is complex and may be country, context, or time dependent; influenced by factors such as levels of socioeconomic inequality and the availability and accessibility of health services (Kelly et al., 2019).

In Aotearoa/NZ, Pacific peoples (a term that refers to 8.1% of the population who have migrated or descended from earlier migrants from nations throughout the Pacific) experience significant and long-standing health inequities (Statistics New Zealand and Ministry of Pacific Island Affairs, 2011). There is evidence that suggests Pacific children in Aotearoa/NZ are less likely to receive an autism diagnosis compared to European children, and those who are diagnosed, are more likely to have higher needs (Bowden et al., 2020; Eggleston et al., 2019; Ruhe et al., 2022; Simpson et al., 2018; Virues-Ortega et al., 2017). It stands to reason that the lower rate of diagnosed autism among Pacific children is likely to be an underestimate of the true prevalence. While there are no current Pacific focussed autism diagnosis services, there are Pacific clinicians who work in the mental health system, and there is at least one Pacific parent support network for autistic Pacific children that has provided many benefits to Pacific families with autistic children (Nafatali, 2023).

It has been reported that Pacific children are less likely to leave school with a formal qualification than non-Pacific children in Aotearoa/NZ (Ministry of Pacific Peoples, 2016). Yet, Pacific peoples with higher levels of education, compared to those with lower qualifications, perceive fewer barriers to accessing healthcare and navigating health systems (Lilo et al., 2020). It may be reasonable to hypothesise that the lower rate of autism could be in part due to fewer educated parents in Pacific families. The primary aim of this study was to investigate the extent to which levels of parents’ formal education is associated with obtaining an autism diagnosis for their Pacific child in Aotearoa/NZ. In addition, we sought to explore the influence of other social, cultural, and economic factors on receiving an autism diagnosis. In this article, identity-first language is used in recognition that this is the preference among many autistic people in Aotearoa/NZ (Monk, 2022). However, to the authors acknowledge, there is no single term preferred by all autistic people.

## Method

### Study design

This was a national cohort study using data from Aotearoa/NZ’s Integrated Data Infrastructure (IDI). The IDI is a large database managed by Statistics New Zealand that probabilistically links data from both government and non-government agencies, as well as data sourced from surveys at an individual level using anonymised, unique identifiers (Milne et al., 2019). Strict protocols and approval processes exist for the use of IDI data (Stats NZ, 2017).

This study included a cohort of children aged 0–9 years, and their parents, identified from the 2013 Census, whose autism diagnostic status was tracked until 2018.

### Primary measures

#### Outcome: autism

The study used an established method to identify autism in the IDI (Bowden et al., 2020). The method draws on diagnostic coding captured within the Programme for the Integration of Mental Health Data (publicly funded specialty mental health services), the National Minimum Dataset (hospital admissions), and Socrates (needs assessment information from disability support services). Autism was indicated if a diagnosis code for autism was identified in any of these datasets at any time over a child’s lifetime up to the end of the study period. Thus, the oldest child would have 15 years to receive a diagnosis while the youngest only 5 years.

#### Stratification variable: ethnicity

Ethnicity was identified from the ‘Personal Details’ table in the IDI. Individuals were classified as Pacific if they identified with one or more Pacific ethnic groups. A composite group, non-Māori/non-Pacific (NMNP), was created as a reference. Individuals with Māori ethnicity were excluded from this study unless also identifying as Pacific. As indigenous peoples of Aotearoa/NZ, Māori command a unique position and are deserving of independent research. Māori also share a heritage with peoples from the Pacific and therefore their presence in a comparison cohort would moderate many characteristics inherent in Pacific.

#### Exposure variable: parental education

The level of parental education was represented by an approximation of the number of years of formal education undertaken by each parent. For multi-parent households, the number of years was averaged across parents. Years of education were determined using the highest self-reported qualification from the 2013 Census. The years of each parent’s education was estimated by transforming the highest qualification level from Aotearoa/NZ’s National Qualifications Framework into an estimated number of years to attain that qualification (an ordinal measure ranging from 0 for no qualification to 11 for a doctoral qualification). International qualifications were acknowledged; however, they were translated to Aotearoa/NZ equivalent qualifications.

#### Other sociodemographic, social, cultural, and economic covariates

Other variables available in the IDI either known, or hypothesised, to associate with a child’s autism diagnosis were used in the models. These variables were chosen to align with the World Health Organization’s social determinants of health (World Health Organization, 2008). These included: child’s sex (male/female); parents’ age, a binary variable indicating young parents who were both 30 years or younger at the time of child’s birth; parents’ place of birth indicating whether any of a child’s parents were born in Aotearoa/NZ; quintile of household income, equivalised to reflect the total income for the household adjusted for the number of people living in the home; level of local area socioeconomic deprivation (also in quintiles); an indicator for single parent; home ownership; Christian religious affiliation; employment; whether conversational English was spoken at home; and whether there were additional adults in the home (individuals 18 years and over).

### Procedure

Data was accessed from the January 2020 refresh of the IDI, extracted and analysed using SAS 7.1 (SAS Institute Inc., 2014). In accordance with Statistics New Zealand protocols, all counts were supressed if less than six and randomly rounded to base three. All statistical outputs were independently reviewed by Statistics New Zealand before being released from the IDI environment. The research adhered to guidelines for observational studies that used administrative data (Benchimol et al., 2015).

### Community involvement

We recognise that engagement with the autistic and autism communities and co-production of knowledge is important to autism research. To this end, the research team included two autistic co-authors (one who is Pacific) and four parents of autistic children (three who are Pacific). In addition, two co-authors are Pacific paediatric clinicians, who have substantial experience working with autistic Pacific children and their families. All have contributed to the study design, drafting, and/or provided critical review of this manuscript.

Given a paucity of evidence about autism in Pacific communities, in some instances interpretation of findings has been informed by the lived experiences of Pacific members of our research team who are from the autism and autistic communities. Their input has added insight, validity and nuance to the quantitative findings reported.

### Statistical analysis

Observed autism status among the 2013 national cohort was examined descriptively, by covariates in the study. Logistic regression models were employed to examine the crude and adjusted association between years of parents’ education and autism among Pacific children. Adjusted models included all sociodemographic, social, cultural, and economic covariates described above. The models were assessed with the Hosmer and Lemeshow goodness of fit test and the c-stat or concordance statistic (Hosmer et al. 2013). Effect sizes are expressed as odds ratios and reported with 95% confidence intervals (CI). Two-tailed α = 0.05 were used to determine statistical significance. Analysis on the NMNP cohort was undertaken for reference and included in supplementary material.

## Results

### Participant population

There were 394,014 children aged 0–9 years identified in the 2013 Census. After excluding 84,312 (21.4%) children who identified as Māori but not also Pacific, 309,702 remained, of whom 49,263 (15.9%) were Pacific and 260,439 (84.1%) were NMNP.

### Demographic description of Pacific children

Overall, 546 Pacific children were identified as autistic (or 1.1% of the Pacific children). In contrast, 4053 NMNP children (1.6%, see supplementary Table 1) were identified as autistic reflecting significant ethnic differences in the incidence of diagnosed autism (Chi-square (df = 1) = 61, *p* < 0.0001).

Compared with non-autistic Pacific children, autistic children had parents with slightly longer educational study (see Table 1). They were nearly 2 years older, less likely to be female, more likely to have fathers born in Aotearoa/NZ, and more likely to live with adults other than their parents.

**Table 1.** Descriptive analysis of regression variables for the total Pacific child sample and Pacific autistic children.

	All Pacific children in this study		Autistic Pacific children		*P* value^a^
	N	(%)	N	(%)	
All children					
Total	49,263	(100)	546	(100)	
Child’s sex					
Male	25,296	51.35	444	81.32	<0.0001
Female	23,967	48.65	102	18.68	
Parent age at child’s birth					
<30 years	17,031	34.57	198	36.26	0.1826
30+ years	32,232	65.43	348	63.74	
Parents born in Aotearoa/NZ					
Neither parent	13,275	26.95	75	13.74	<0.0001
At least one parent	35,991	73.05	471	86.26	
Equivalised household income quintile					
Lowest income	17,772	36.08	204	37.16	0.3505
Quintile 2	12,876	26.14	150	27.32	
Quintile 3	8,472	17.20	90	16.39	
Quintile 4	6,168	12.52	63	11.48	
Highest income	3,972	8.06	42	7.65	
NZDep2013 quintile					
Least deprived	3,165	6.43	36	6.59	0.2377
Quintile 2	4,629	9.40	60	10.99	
Quintile 3	6,774	13.75	81	14.84	
Quintile 4	10,701	21.72	120	21.98	
Most deprived	23,991	48.70	249	45.60	
Sole parent					
One parent	15,939	32.35	213	38.80	0.0006
Two parents	33,324	67.65	336	61.20	
Home ownership					
Rented	37,554	76.23	420	76.50	0.3555
Owned	11,712	23.77	129	23.50	
Christian church affiliated					
Both parent	22,263	45.19	207	37.91	0.0004
One parent	14,607	29.65	174	31.87	
Neither parent	12,396	25.16	165	30.22	
Parents employed					
At least one parent	37,077	75.26	387	70.88	0.0084
Neither parent	12,186	24.74	159	29.12	
English spoken					
At least one parent	36,174	73.43	402	73.63	0.3678
Neither parent	13,089	26.57	144	26.37	
Additional adults in household					
Other adults	40,743	82.71	414	75.82	<0.0001
Parents only	8,520	17.29	132	24.18	
	Mean	(95% CI)	Mean	(95% CI)	*p* value^b^
Parents’ highest education qualification					
Years to highest qualification	2.38	(2.36, 2.40)	2.56	(2.41, 2.72)	<0.0001

CI = confidence interval.

a *p* value is reported for a chi-square test of categorical variables for the differences between children with autism versus those without. ^b^p value is reported for a *t*-test of means for the differences between children with autism versus those without.

### Association between parents’ education and autism in Pacific children

The baseline univariate model showed that there is an approximately 8% increased odds of receiving an autism diagnosis for their Pacific child for each additional year of parents’ education (odds ratio (OR) = 1.08; 95% CI = 1.04–1.12, *p* = 0.0007; see Table 2).

**Table 2.** OR together with 95% CI for influence of covariates on children’s odds of autism identification among Pacific children.

Covariate	Unadjusted		Adjusted	
	OR	95% CI	OR	95% CI
Parents’ education				
per year for parents’ highest qualification	1.079	[1.04–1.12]	1.102	[1.06–1.15]
Child’s sex				
Male			Reference	
Female			0.228	[0.19–0.27]
Parent age at child’s birth				
<30 years			1.001	[0.84–1.19]
30+ years			Reference	
Parents born in Aotearoa/NZ				
Both parents			Reference	
Father only			0.947	[0.78–1.16]
Mother only			1.021	[0.80–1.31]
Neither parent			0.377	[0.30–0.48]
Equivalised household income quintile				
Quintile 1 (lowest income)			Reference	
Quintile 2			1.014	[0.83–1.24]
Quintile 3			0.933	[0.74–1.18]
Quintile 4			0.752	[0.57–0.99]
Quintile 5 (highest income)			0.701	[0.51–0.96]
NZDep2013 quintile				
Quintile 1 (least deprived)			0.982	[0.72–1.34]
Quintile 2			1.287	[1.01–1.64]
Quintile 3			1.082	[0.87–1.35]
Quintile 4			1.134	[0.94–1.37]
Quintile 5 (most deprived)			Reference	
Sole parent				
One parent			1.116	[0.88–1.41]
Two parents			Reference	
Home ownership				
Owned			Reference	
Rented			0.943	[0.79–1.13]
Christian church affiliated				
Both parents			Reference	
One parent			0.982	[0.81–1.19]
Neither parent			0.984	[0.81–1.20]
Parents employed				
At least one parent			0.697	[0.57–0.85]
Neither parent			Reference	
English spoken				
At least one parent			1.373	[1.07–1.76]
Neither parent			Reference	
Additional adults in household				
Other Adults			0.687	[0.54–0.87]
Parents only			Reference	

OR: odds ratio; CI: confidence interval.

After adjustment, controlling for a range of sociodemographic, cultural, social, and economic factors, parents’ education remained significant, with the effect size strengthening slightly to reflect an increased odds of receiving an autism diagnosis to 10% for each additional year of parents’ education (OR = 1.10; 95% CI = 1.06–1.15, *p* = 0.0007).

### Other factors associated with autism identification in Pacific children

A number of other covariates in the model were also significantly related to autism including sex (female vs male children) (OR = 0.23; 95% CI = 0.19–0.27, *p* ⩽ 0.0001), neither parent born in Aotearoa/NZ (OR = 0.38; 95% CI = 0.30–0.48, p ⩽ 0.0001), at least one employed parent (OR = 0.70; 95% CI = 0.57–0.85, *p* = 0.03), English fluency (OR = 1.37; 95% CI = 1.07–1.76, *p* < 0.05), and additional adults in the house (OR = 0.69; 95% CI = 0.54–0.87, *p* = 0.03). Thus, the odds of identifying autism in Pacific children was significantly lower if at least one parent was employed, neither parent was born in Aotearoa/NZ, or if other adults lived in their house and higher if conversational English was regularly spoken at home.

Both unadjusted (chi-sq = 8.6, *p* = 0.38) and adjusted (chi-sq = 12.9, *p* = 0.07) models tested satisfactorily by the Hosmer and Lemeshow test. The unadjusted model was only weakly predictive of autism (C-stat = 0.54, 95% CI = 0.52–0.56) while the adjusted model was satisfactorily predictive (C-stat = 0.73, 95% CI = 0.71 to 0.75).

## Discussion

### Key findings

#### Associations between parents’ education and autism within Pacific children

This nationwide cohort study is the first to examine the association between the level of parental education and autism among Pacific children. Our findings show that Pacific children are 30% less likely to receive an autism diagnosis compared with NMNP children in Aotearoa/NZ but that increased parental education is significantly associated with increased identification of autism in Pacific children. This association with parental education persists even after adjusting for a range of social, economic, and cultural factors. The finding that higher levels of parental education is associated with increased identification of autism is consistent with some international evidence (Dickerson et al., 2017; Durkin et al., 2010; Fountain et al., 2011; Kelly et al., 2019; Thomas et al., 2012), but is in contrast to others (Larsson et al., 2005; Maenner et al., 2023; Rai et al., 2012; Roman-Urrestarazu et al., 2021). The association between parental education and autism diagnosis could be due to increased autism awareness. Parents with higher levels of education may acquire an autism diagnosis by having the capability to first; identify signs of autism and second; articulate those signs in a non-Pacific healthcare or education system. However, while higher education may improve a parent’s ability to recognise symptoms of autism, any parent who has not previously been exposed to autism may still not identify it. Thus, awareness of autism is an important part of the health literacy journey for parents and all members of Pacific communities, an area of research that has not been thoroughly explored in those communities (Apulu-Pamatatau, 2022; Masi, 2022).

Obtaining an autism diagnosis for a child is a complex process and more so in families who have difficulties negotiating unfamiliar health settings (Eggleston et al., 2019). Moreover, recent evidence suggests Pacific peoples with higher levels of education have lower barriers to accessing healthcare and navigating health systems (Lilo et al., 2020). For example, in a recent study, higher parental education was associated with lower incidence of potentially avoidable hospitalisations in Pacific children (Kokaua et al., 2023). Therefore, shortcomings in the health system to provide accessible services to all Pacific families may also explain lower rates of autism diagnosis among those families with lower levels of formal education.

#### Influence of other factors on autism in Pacific children

Our findings also suggest that in addition to education, other parental factors including being born in Aotearoa/NZ and speaking English at home were associated with increased odds of autism identification. These factors are correlated with parental education and therefore likely also indicate higher levels of health literacy and ability to navigate a Western health system. However, they may also be indicative of higher levels of European acculturation and reflect several cultural intricacies. For example, parents with higher levels of acculturation may feel less burdened by the social stigma (including heightened feelings of shame and anxiety) of seeking mental health support and a diagnosis for their child which can be common among Pacific communities (Fa’alogo-Lilo & Cartwright, 2021). However, evidence shows that when a family or community member has been diagnosed, their communities can also become more supportive through gained improved awareness of such conditions (Apulu-Pamatatau, 2022).

In addition, traditionally autism might be viewed as the result of a spiritual transgression or imbalance (Capstick et al., 2009). Thus, Pacific parents with more intrenched cultural values might be more likely to accept the child’s differences and not pursue a medical diagnosis (Ministry of Health, 2020). Finally, traditionally Pacific people adhere to a family of community-centred approach to caring for their children. Consequently, the primary responsibility for childcare can rest within the confines of family and community, with medicalised help sough only if it aligns with Pacific cultural values and practices.

Surprisingly, employment and higher incomes were also associated with lower odds of autism in Pacific children. This is in contrast with findings that showed Pacific people’s health literacy, service accessibility and health behaviours were positively associated with socioeconomic status (Sa’uLilo et al., 2018). While it is evident that autistic children are more likely to live in families with neither parent employed, international studies have not clearly determined which direction a causal relationship may take for income or employment (Dickerson et al., 2017; Durkin et al., 2010; Fountain et al., 2011; Maenner et al., 2023; Roman-Urrestarazu et al., 2021; Thomas et al., 2012). Understanding the relationships between a range of socioeconomic factors and obtaining an autism diagnosis is complex and requires further research to fully explain their association with attaining a diagnosis for Pacific children in Aotearoa/NZ.

While religious affiliation had no association with autism in Pacific children, spirituality is common in most Pacific communities, nearly two-thirds of Pacific autistic children in this study have at least one parent who is affiliated with a Christian belief, and it is central in many understandings of health (Pulotu-Endemann, 2009). It is not uncommon for some parents to believe their children’s behaviour may be a result of breaches in tapu (sacredness) requiring traditional modes of healing (Capstick et al., 2009). There is some debate about the benefits or otherwise of religion to Pacific communities (Teevale et al., 2016).

#### Implications from these findings

It is well-documented that early support for autistic children and their families offers numerous short- and long-term benefits (Whitehouse et al., 2021) and that receiving this support is facilitated by receiving an early diagnosis. Therefore, it is crucial that a timely and equitable diagnostic process exists for Pacific children. The absence of support places autistic children at greater risk of a range of academic, social, and behavioural difficulties (Lang et al., 2010, 2013; Watkins et al., 2015). Considering the findings of this study, more must be done to facilitate access to diagnostic services for all Pacific families, particularly those with lower levels of formal education. However, equitable access to diagnostic services will only be beneficial if support and treatment services are accessible to this population as well. Over the past decade, there has been a growing recognition that the current health system has not met the needs of Pacific peoples (Ministry of Health, 2020).

Ultimately, the burden of care for families with autistic children has been assumed by the Pacific communities themselves. A new and innovative approach is required to ensure greater awareness of autism and that those communities are resourced to provide sustainable and appropriate care for families of autistic children. At the very least, recommendations pertaining to Pacific populations from the Aotearoa New Zealand Autism Guideline: He Waka Huia Takiwātanga Rau should be pursued (Whaikaha–Ministry of Disabled People & Ministry of Education, 2022). These include: ensuring that appropriate information is provided that takes both language and culture into account so that Pacific communities are aware of the support services they are eligible for (recommendation 8.2); making certain that services are proactive in offering supports (8.5); targeted recruitment strategies to improve the capability and capacity of the Pacific autism-related workforce (8.8); and improving the cultural competency of the existing health workforce (8.9).

#### Strengths and limitations

The study has a number of strengths. Most notably, its novel use of IDI data which facilitates the linkage of health and non-health data, as well as child-to-parent data. This meant we were able to estimate the association between a parent’s education and their child receiving an autism diagnosis, while controlling for a range of child and parent characteristics. The IDI also enables the identification of a national cohort of Pacific children resulting in a sample size large enough to undertake such analyses. Furthermore, the IDI contains more robust ethnicity data sourced from multiple datasets, which is an improvement over using ethnicity captured in any single data collection.

In addition, the recent edition of Aotearoa New Zealand Autism Guideline: He Waka Huia Takiwātanga Rau highlights the void in Pacific autism research and includes a recommendation 8.7 that research streams be established to provide baseline information on autism and Pacific peoples (Whaikaha–Ministry of Disabled People & Ministry of Education, 2022). Moreover, internationally, over 85% of cases of autism in epidemiological studies are identified from only 10% of the world’s children, mostly from North America, Europe, and Japan (Barbaro & Halder, 2016). Therefore, this study offers a timely contribution to an understudied population in the field of autism research.

However, the findings must also be considered in the context of several limitations. First, the study used an autism case identification method that, while novel, is yet to be validated. The extent to which the method produces false autism identifications and undercounts autism among Aotearoa/NZ children is unknown. Another limitation is that the method of identifying those as autistic is dependent upon health service interactions and may bias towards capturing higher need cases of autism, particularly among Pacific children (Ruhe et al., 2022).

## Conclusion

This study shows parents’ level of formal education is associated with better identification of autism among Pacific children. The findings suggest that higher education may help enable parents to better navigate the health system. However, they also suggest ongoing systemic failure of the health system to appropriately cater for needs of Pacific communities.

## Supplemental Material

sj-docx-1-aut-10.1177_13623613231217800 – Supplemental material for Is parent education a factor in identifying autism/takiwātanga in an ethnic cohort of Pacific children in Aotearoa, New Zealand? A national cross-sectional study using linked administrative data Supplemental material, sj-docx-1-aut-10.1177_13623613231217800 for Is parent education a factor in identifying autism/takiwātanga in an ethnic cohort of Pacific children in Aotearoa, New Zealand? A national cross-sectional study using linked administrative data by Jesse Kokaua, Betty Kolose-Pulefolau, Troy Ruhe, Faith Aldridge, Siale Foliaki, Liam Kokaua, Talai Mapusua, Joanne Dacombe, Rosalina Richards, Russell Blakelock and Nicholas Bowden in Autism
