# Supplementary material for: Is parent education a factor in identifying autism/takiwātanga in an ethnic cohort of Pacific children in Aotearoa, New Zealand? A national cross-sectional study using linked administrative data
Source: Autism. 2023 Dec 28;28(7):1667–76. doi: 10.1177/13623613231217800 (PMC11191371; doi:10.1177/13623613231217800)
Supplement: sj-docx-1-aut-10.1177_13623613231217800 – Supplemental material for Is parent education a factor in identifying autism/takiwātanga in an ethnic cohort of Pacific children in Aotearoa, New Zealand? A national cross-sectional study using linked administrative data [file sj-docx-1-aut-10.1177_13623613231217800.docx]

**Supplementary Table 1**. Descriptive analysis of regression variables for the NMNP child cohort and NMNP autistic children.

|  |  | **All NMNP children in this study** | | **Autistic NMNP children** | | **p-value^1^** |
| --- | --- | --- | --- | --- | --- | --- |
|  |  | **N** | **(%)** | **N** | **(%)** |  |
| All children | **Total** | **260,436** | **(100%)** | **4053** | **(100%)** |  |
| Parents’ highest education qualification | Mean years  (95% CI) | 3.92 | (3.91,3.93) | 3.80 | (3.73,3.86) | <0.0001 |
| Child’s sex | Male | 133608 | 51.30% | 3210 | 79.20% | <0.0001 |
|  | Female | 126828 | 48.70% | 843 | 20.80% |  |
| Parent age at child’s birth | <30 years | 38541 | 14.80% | 624 | 15.40% | <0.0001 |
|  | 30+ years | 221898 | 85.20% | 3429 | 84.60% |  |
| Parents born in Aotearoa/NZ | Neither parents | 61044 | 23.44% | 867 | 21.38% | <0.0001 |
|  | At least one parent | 199392 | 76.56% | 3189 | 78.62% |  |
| Equivalised household income quintile | Lowest income | 39399 | 15.13% | 774 | 19.11% | <0.0001 |
|  | Quintile 2 | 57513 | 22.08% | 1035 | 25.56% |  |
|  | Quintile 3 | 57558 | 22.10% | 888 | 21.93% |  |
|  | Quintile 4 | 54138 | 20.79% | 702 | 17.33% |  |
|  | Highest income | 51834 | 19.90% | 651 | 16.07% |  |
| NZDep2013 quintile | Least deprived | 70941 | 27.24% | 903 | 22.23% | <0.0001 |
|  | Quintile 2 | 61653 | 23.67% | 891 | 21.94% |  |
|  | Quintile 3 | 56469 | 21.68% | 912 | 22.45% |  |
|  | Quintile 4 | 44931 | 17.25% | 771 | 18.98% |  |
|  | Most deprived | 26445 | 10.15% | 585 | 14.40% |  |
| Sole parent | One Parent | 33213 | 12.75% | 780 | 19.23% | <0.0001 |
|  | Two Parent | 227226 | 87.25% | 3276 | 80.77% |  |
| Home ownership | Rented | 89919 | 34.53% | 1707 | 42.12% | <0.0001 |
|  | Owned | 170517 | 65.47% | 2346 | 57.88% |  |
| Christian church affiliated | Both parents | 68772 | 26.41% | 1032 | 25.44% | <0.0001 |
|  | One parent | 57786 | 22.19% | 945 | 23.30% |  |
|  | Neither parent | 133881 | 51.41% | 2079 | 51.26% |  |
| Parents employed | At least one | 242490 | 93.11% | 3633 | 89.64% | <0.0001 |
|  | Neither parent | 17949 | 6.89% | 420 | 10.36% |  |
| English spoken | At least one parent | 236721 | 90.89% | 3567 | 87.94% | <0.0001 |
|  | Neither parent | 23718 | 9.11% | 489 | 12.06% |  |
| Additional adults in household | Other Adults | 235956 | 90.60% | 3492 | 86.09% | <0.0001 |
|  | Parents only | 24483 | 9.40% | 564 | 13.91% |  |

^1^ p-value is reported for a t-test with averages and chi-squared test with tabulated cell counts for the differences between children with autism versus those without.

**Supplementary Table 2**. Odds ratios, together with 95% CI, for influence of covariates on children’s odds of autism identification among NMNP children

|  |  | Unadjusted | | Adjusted | |
| --- | --- | --- | --- | --- | --- |
| Covariate |  | **Odds ratio** | **95% CI** | **Odds ratio** | **95% CI** |
| Parents’ education | per year for parents’ highest qualification | 1.013 | [1.002 to 1.025] | 1.072 | [1.06 to 1.09] |
| Child’s sex | Male |  |  | Reference |  |
|  | Female |  |  | 0.296 | [0.28 to 0.31] |
| Parent age at child’s birth | <30 years |  |  | 0.691 | [0.63 to 0.75] |
|  | 30+ years |  |  | Reference |  |
| Parents born in Aotearoa/NZ | Both parents |  |  | Reference |  |
|  | Father only |  |  | 1.228 | [1.14 to 1.32] |
|  | Mother only |  |  | 1.051 | [0.97 to 1.14] |
|  | Neither parent |  |  | 0.822 | [0.77 to 0.88] |
| Equivalised household income quintile | Quintile 1 (lowest income) |  |  | Reference |  |
|  | Quintile 2 |  |  | 1.127 | [1.05 to 1.22] |
|  | Quintile 3 |  |  | 0.918 | [0.85 to 1.00] |
|  | Quintile 4 |  |  | 0.758 | [0.70 to 0.83] |
|  | Quintile 5 (highest income) |  |  | 0.623 | [0.57 to 0.68] |
| NZDep2013 quintile | Quintile 1 (least deprived) |  |  | 0.779 | [0.72 to 0.85] |
|  | Quintile 2 |  |  | 0.815 | [0.75 to 0.89] |
|  | Quintile 3 |  |  | 0.833 | [0.77 to 0.90] |
|  | Quintile 4 |  |  | 0.847 | [0.78 to 0.92] |
|  | Quintile 5 (most deprived) |  |  | Reference |  |
| Sole parent | Yes |  |  | 1.209 | [1.1 to 1.33] |
|  | No |  |  | Reference |  |
| Home ownership | Owned |  |  | Reference |  |
|  | Rented |  |  | 0.797 | [0.75 to 0.84] |
| Christian church affiliated | Both parents |  |  | Reference |  |
|  | One parent |  |  | 1.057 | [0.99 to 1.13] |
|  | Neither parent |  |  | 1.018 | [0.96 to 1.08] |
| Parents employed | At least one parent |  |  | 0.666 | [0.61 to 0.73] |
|  | Neither parent |  |  | Reference |  |
| English spoken | At least one parent |  |  | 1.543 | [1.41 to 1.69] |
|  | Neither parent |  |  | Reference |  |
| Additional adults in household | Other Adults |  |  | 0.782 | [0.71 to 0.86] |
|  | Parents only |  |  | Reference |  |
